# Interpersonal symptoms in adolescence depression across Asian and European regions: a network approach

**DOI:** 10.1186/s12888-024-06161-9

**Published:** 2024-10-22

**Authors:** H. N. Cheung, M. Habibi Asgarabad, W. S. Ho, M. R. Zibetti, S. T. K. Li, W. Y. Chan Stella, J. M. Williams

**Affiliations:** 1 Department of Social Work and Social Administration, The University of Hong Kong, Pokfulam, Hong Kong SAR China; 2 Department of Psychology, Norwegian University of Science and Technology, Norges Teknisk-Naturvitenskapelige Universitet, Trondheim, Norway; 3 Department of Social and Behavioural Sciences, City University of Hong Kong, Hong Kong SAR, China; 4 Universidade do Vale do Rio dos Sinos, Sao Leopoldo, Brazil; 5 School of Arts and Social Sciences, Hong Kong Metropolitan University, Hong Kong SAR, China; 6 Charlie Waller Institute of Evidence-Based Psychological Treatment, University of Reading, Reading, UK; 7 Department of Clinical and Health Psychology, University of Edinburgh, Edinburgh, UK

**Keywords:** Depression, Adolescents, Interpersonal Symptoms, Network model

## Abstract

**Background:**

Major Depressive Disorder (MDD) poses a significant global health challenge, with symptom presentation potentially varying between adolescents and adults. Adolescence is a critical period marked by heightened vulnerability to interpersonal stresses, yet the impact of these stresses on the structure of depressive symptoms is not well understood. Recognizing the cultural nuances in how depression manifests among adolescents is crucial. To this end, this paper employs a network analysis approach, utilizing a comprehensive symptom checklist from the Multidimensional Depression Assessment Scale (MDAS). Our study investigates the role of interpersonal symptoms within the broader cluster of emotional, cognitive, and somatic symptoms and explores variations in adolescent groups in four Asian and European regions.

**Methods:**

We recruited a diverse sample of 6,348 adolescents aged 12 to 18 from Hong Kong, Taiwan, the UK, China, and the Netherlands using the Qualtrics platform. Employing the Gaussian Graphical Model, we established a network model of depressive symptoms as measured by the MDAS, segregating the sample into Asian and European regions to examine the interconnections between them. The study focused on identifying central symptom nodes and comparing the network structures between the two groups.

**Results:**

The analysis identified *feeling worthless*,* low energy*,* being a burden to others*, and *low mood* as central symptoms of depression. Notably, there were significant differences in the connections between depressive symptoms among Asian (Hong Kong, China and Taiwan) and European (UK and the Netherlands) adolescents, highlighting cultural variations in how interpersonal symptoms interact with emotional, cognitive, and somatic symptoms.

**Conclusion:**

This study is pioneering in applying network analysis to include interpersonal symptoms in examining depression among a diverse adolescent population. It demonstrates that interpersonal symptoms are integral to the central features of depressive symptoms. Furthermore, our findings suggest that, compared to their UK and Dutch peers, interpersonal symptoms in Asian adolescents are uniquely connected to other symptom clusters, reflecting distinct cultural patterns. Limitations: The study engaged a broad community sample; however, future research could benefit from including a larger sample size to allow for a more detailed analysis of a greater number of symptom nodes.

**Supplementary Information:**

The online version contains supplementary material available at 10.1186/s12888-024-06161-9.

## Introduction

Major Depressive Disorder (MDD) affects 300 million individuals of all ages around the globe [1]. It has a prevalence of 4–5% in middle to late adolescence [2]. Numerous studies on cultural differences in depression compared Asians and Westerners in terms of their self-reported ethnicity [3, 4]. MW Flores, A Sharp, NJ Carson and BL Cook [5] reported a lower prevalence of MDD in Asian (14.6% [95% CI, 9.6-19.5%]) adolescents than in white adolescents (20.2% [95% CI, 18.3-22.2%]). The clinical presentation and cause of major depressive disorder (MDD) vary widely [6]. Interpersonal features, including feeling inferior and feeling biasedly perceived, have been shown to be central transdiagnostic traits and disorders prior to treatment [7]. The interpersonal model [8–12] considered mental illness as an individual’s social maladaptation. Inadequate interpersonal communication triggers and maintains mental illness [13]. The way depressive symptoms are displayed may differ depending on cultural backgrounds through interpersonal symptoms [14, 15]. AJ Umaña-Taylor and KA Updegraff [16] discovered significant variations in the prevalence of depression among various racial and ethnic groups and pointed to cultural variations in how depressive symptoms, including interpersonal aspects, are manifested. In addition, an interpersonal model of adolescent depression by Cohen et al. considers grief, interpersonal conflicts, role changes, and social isolation as the factors contributing to the development of depression. The study emphasizes the importance of cultural context in comprehending the interpersonal aspects of depression [17]. In the Chinese population, depression manifests as neurasthenia and somatization [18]. Similarly, in Japan, depression was predominantly linked to individual behavioural and psychological characteristics [19].

Recent data has additionally emphasized the portrayal of interpersonal symptoms in depression across different cultures. A systematic review of 170 study populations representing 76 nationalities found the most reported depressive symptoms and features across different cultures. The study revealed that social isolation/loneliness was one of the most frequently observed features of depression, in addition to the symptoms identified by the Diagnostic and Statistical Manual of Mental Disorders (DSM) diagnostic criteria [20]. Trauma-affected individuals often exhibit interpersonal difficulties, such as a hesitancy to engage in conversation and a heightened sense of suspicion, at a higher frequency compared to other communities. The findings reveal that the diagnostic criteria of Major Depressive Disorder (MDD) disregarded the crucial role of interpersonal symptoms in the cluster of depression [13]. Furthermore, interpersonal manifestations of depression are also supported by primarily assigning interpersonal explanations to depression rather than cognitive causes in Nepali teenagers [10]. The heart-mind ethnopsychology, as observed in Nepalese culture about emotions, experiences, and memories [9] is well recognized and prevalent across several Asian countries. Despite their importance, interpersonal symptoms are absent due to the intrapersonal qualities of diagnostic criteria and screening tools [21]. However, prior research has not been as thorough in exploring interpersonal symptoms in the cultural adaptation of depression among adolescents. And a majority of studies focused on gender differences in interpersonal stressors and depressive symptoms [22].

As a result, relying merely on sum or mean scoring that provides equal weight to all symptoms may lead to an inaccurate measure of depression severity [23, 24]. As the most prevalent symptom profile was only validated by 1.8% of teenage outpatients, the highly heterogeneous symptom pattern presents significant challenges in intervention design.

Traditional psychopathology implies that symptoms co-occur due to underlying depression [25]. Yet an emerging perspective predicates the idea that mental disorders may trigger and intensify one another [26]. Symptoms may also reinforce one another by forming a feedback loop or leading to several reciprocal interactions. These interconnections may cause a constellation of symptoms. In light of these, the network method, which overcomes the constraints of the present average level of symptoms and pinpoints the defining characteristics of the depressed experience, has emerged in popularity [26]. Each symptom is represented as a node in a network, and the nodes are linked to one another by lines of varied thickness (edges). The likelihood that two symptoms activate each other via biological, psychological, or other processes is represented by the strength of the statistical correlation found between them, which is represented by the edge’s thickness. Sparse symptom networks are designed to highlight just the most important relationships between symptoms [27]. The central nodes represent the most interrelated symptoms. It is generally agreed that core symptoms play a pivotal role in both the development and maintenance of a syndrome because they are the ones most likely to trigger subsequent symptoms. Conventional centrality indices such as strength centrality have been reported to fail to differentiate between positive and negative connections and may not effectively evaluate the characteristics and intensity of a node’s impact inside the network [28]. Yet the expected influence centrality index has been shown to overcome the limitations. It could lead to important predictions about the nodes that lead to effective treatments [29].

While the network model of depression utilizing the Patients Health Questionnaire (PHQ-9) has been extensively studied in the current literature [30], it may not adequately capture the interplay between interpersonal symptoms and other components of depressive symptoms, which may contribute to a distinct symptom network in adolescents [31]. Mullarkey et al. [32] established a network model based on a US adolescent community sample. Yet a comparison across cultural groups is lacking. The difference between network models across cultural groups allows for the examination of how symptoms of depression dynamically interact within different cultural contexts. It provides an alternative way to characterize and understand the cultural impact on the nature of the disorder [33]. The cultural-specific connections capture the heterogeneity of interrelated symptoms, thereby fostering more effective treatments [34]. As a result, the current paper aimed to establish network models of depressive symptoms across Hong Kong, China, and Taiwan, UK and the Netherlands in a large, diverse sample of adolescents. We collected the data through two large cross-cultural studies on the mental health of adolescents. This project is the first to compare across Eastern and Western ethnic groups using the MDAS, which contains a comprehensive subscale of interpersonal symptoms. Rather than focusing on the ethnic minorities in a particular nation, its sample spans across five regions of the world on a platform of diversity in humanity. The present study provided a methodological advantage over previous research, which has typically sourced ethnic groups from a single nation by emphasizing self-reported ethnicity over nationality. This approach to self-identification aligns closely with an individual’s sense of cultural lineage, encompassing language, societal practices, customs, and the geopolitical context with which they associate. Utilizing self-reported ethnic categories is likely to yield more precise comparisons between cultural groups, as these categories tend to reflect more uniform populations than those based on national distinctions. H Tang, T Quertermous, B Rodriguez, SL Kardia, X Zhu, A Brown, JS Pankow, MA Province, SC Hunt, E Boerwinkle, et al. [35] found a substantial agreement between individuals’ self-identified ethnicity and their genetic cluster affiliations. Evidence also suggests that self-classifications of ethnicity (such as white/European and Asian) often accurately reflect individuals’ ancestral origins [36].

The result would greatly address the knowledge gap on the role and cultural manifestation of interpersonal depression in adolescents. It also aligned with the strong supporting evidence for the continuous nature of depression [37]. The current study examined the central symptoms of depression and compared the role of interpersonal symptoms and their connections to other symptoms across Asian regions of Hong Kong, China, and Taiwan and European regions of UK and the Netherlands in community samples. Hence, a network structure from a community sample has clinical implications for community wellbeing and treatment planning.

## Methods

### Participants and procedures

An adolescent’s community sample of 6348 individuals was recruited through Qualtrics in Hong Kong, China, Taiwan, the United Kingdom, and the Netherlands. The sample came from two large studies on depression in high-ability adolescents, and the areas were under-researched. Yet the five regions from both East Asia regions (Hong Kong, Taiwan, and China) and Europe regions (UK and Netherlands) were included to provide a mix of Eastern and Western cultures, offering a broad spectrum of cultural values, beliefs, and practices for the study of depression. The samples come from a varied pool of countries, potentially increasing diversity [38]. Our study’s sample comprised participants composed of two ethnic backgrounds: white and Asian. All individuals were adolescents, with ages ranging from 12 to 18 years. Before participation, the university ethics committee approved the study, and informed consent was obtained from both the participants and their parents.

To ensure the quality of our data, we implemented a rigorous quality assurance process. We excluded any questionnaires completed in under 5 min, as well as responses from participants who provided illogical answers to fundamental screening questions.

Data collection occurred in two separate phases: the first from June to August 2021, and the second from September to December 2022. The large sample size was deliberately chosen to address the complexities of the network model and produce robust results that could withstand potential inaccuracies in the model’s specification.

### Measures

#### The multidimensional depression assessment scale

Developed by HN Cheung and MJ Power [14], the 52-item multidimensional depression assessment scale examines depressive severity in 4 areas of depressive symptoms on a 5-point Likert scale: emotional, cognitive, interpersonal, and somatic. It has been validated in UK and HK community samples and a clinically depressed and pregnant sample in Inner Mongolia with good psychometric properties, including a Cronbach’s alpha over 0.9 [39]. The scale was widely validated in clinical [40] and non-clinical samples [41] and in adolescents across genders [42] and cultures (Asian, White, Black, and Hispanic) with good validity and a stable factor structure [15]. The back translation process [43] of the Dutch version was adopted and performed by the Language Center of Radboud University.

### Data analysis

All the items on the MDAS were analyzed for their mean, standard deviation (SD), skewness, and kurtosis. We estimated the network using Markov Random Fields, designed to detect conditional relationships between variables. To address the issue of nonnormal item distributions in MDAS, the analyses relied on Spearman correlations. The Gaussian Graphical Model (GGM), which captures conditional relationships among all observed variables while controlling for the linear influence of each other variable, was estimated [44]. The GGM is the most widely employed network model. The thresholded EBICglasso method was utilised to estimate GGMs [45]. The EBICglasso method estimates network models by employing the graphical least absolute shrinkage and selection operator (LASSO) or glasso regularisation technique [46]. To determine the most effective regularisation parameter, the extended Bayesian Information Criterion (EBIC) is adopted [47]. LASSO regularisation typically eliminates false positives by setting small edge weights to precisely zero, which results in a sparser network with fewer edges. The goldbricker function from the networktools package in R [48] checks for multicollinearity (extremely high overlap between nodes) before adding all the nodes to the network model. Its incorporation of self-feedback of anti-trigonometric functions in chaotic neural network models has been observed to impact the network’s optimisation mechanism, hence avoiding the network from becoming stuck in local minima [49]. It systematically evaluates all potential correlations within a psychometric network and establishes the fraction of correlations that are considered to be “insufficiently high”. A selection of the best nodes in a highly correlated pair was retained in the network.

In addition, the degree of centrality of each symptom was estimated to identify symptoms that have strong connections with others [50]. Nodes with high centrality are regarded as having an above-average level of impact on the rest of the network [2]. Expected influence is a measure that considers both the strength and directionality of an edge. It calculates a node’s strength by using the actual values of edge weights rather than their absolute values [28]. In particular, TR Spiller, O Levi, Y Neria, B Suarez-Jimenez, Y Bar-Haim and A Lazarov [51] found that out of the three centrality metrics (strength, predictability, and expected influence), only the expected influence accurately predicted the degree to which changes in nodes/symptoms were associated with changes in other nodes/symptoms. As a result, the expected influence was extensively reported in the current study, which has significant implications for future treatment guidance.

The Fruchterman-Reingold algorithm produced a graphical presentation of the results, in which the central nodes (symptoms) emerged at the center of an optimal layout of a network of symptoms based on closeness [52]. This method inserts nodes with numerous and strong connections prominently, as well as highly linked node pairs near each other, while also producing a visually pleasing layout with no node or edge overlap.

Additionally, we examined the accuracy and stability of the network model using boostrapping procedures described by S Epskamp, D Borsboom and EI Fried [45] and visualized the results using *qgraph* in R [53]. All network estimates were performed using the R program *bootnet* [54]. The correlation stability (CS) coefficient was used. This is a measure of how many instances may be discarded before a correlation of 0.70 for node order for strength centrality between the original network and the new network is computed without violating the dropped cases. A CS co-efficient greater than 0.25 is suggested, while values greater than 0.50 indicate good resilience [45]. The maximum possible value is 0.75. Bootstrapped 95% confidence intervals were also calculated for all edge weights.

Finally, we evaluated network differences between Asian areas of Hong Kong, China, and Taiwan and European areas of UK and the Netherlands samples using the permutation test Network Comparison Test (NCT), which determines the difference between two networks (Asian vs. European participants) [55]. The NCT was performed on gender-defined subsamples with 1000 permutations, as previously advised [56]. The global network strength was determined by comparing the absolute sum of all edge weights between the networks. Following that, the distributions of edge weights within each network were compared in order to determine the network’s structure. The differences in strength for each edge were compared between the two networks (Holm–Bonferroni correction of p values). All tests were run using the R package “NetworkComparisonTest” 2.0.1 [57].

## Results

### Descriptive statistics

A total of 6348 individuals, 3885 from Hong Kong, China and Taiwan and 2463 from UK and the Netherlands, were recruited for the study with a mean age of 16.32 (S.D. = 1.52). A relatively equal distribution of females and males was reported, and 12.78% of participants reported experiencing mental issues in the past two weeks. Table 1 displays the summary of demographic variables. The mean, standard deviation, skewness, and kurtosis of depressive symptoms as evaluated by the MDAS are reported in supplementary Table 1.

**Table 1:** Descriptive statistics of participants’ demographics

	Mean (S.D)
Age	16.32 (1.52)
	n (%)
Gender	
Male	3064 (48.27)
Female	3258 (51.32)
Others	26 (0.41)
History of mental disorders	
No	5537 (87.22)
Yes	811 (12.78)

**Fig. 1:**
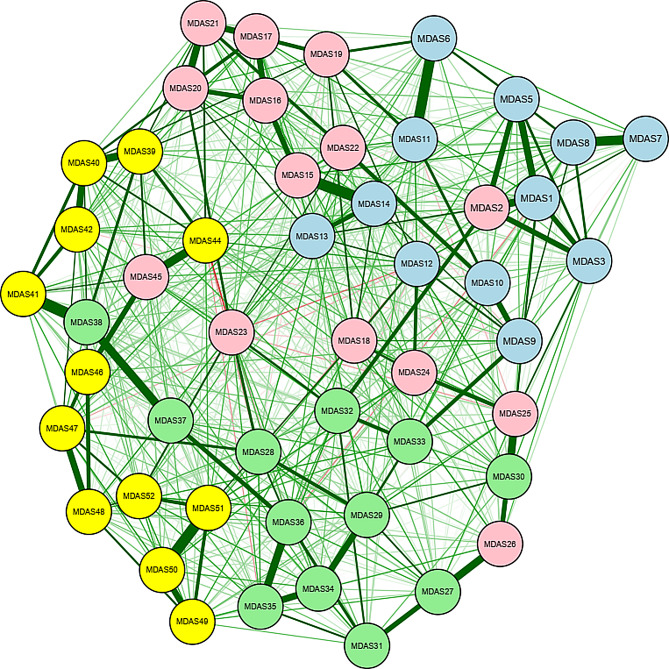
Network structure for MDAS. *Note* Each node represents a variable. The lines represent edge between nodes. Wider and more saturated edges indicate stronger associations. All associations are positive. Only 50 nodes out of 52 items are significantly connected. The nodes are colour-coded MDAS1-12 in blue, MDAS13-24, MDAS49-51 in red, MDAS25-36 in green, and MDAS 37–48 in yellow Emotional domain: MDAS1. Low mood MDAS2. Sadness MDAS3. Low spirits MDAS4. Gloominess MDAS5. Sad mood MDAS6. Guilt MDAS7. Unhappiness MDAS8. Not cheerful MDAS9. Irritable mood MDAS10. Dysphoric mood MDAS11. Shame MDAS12. Anxiety Cognitive domain: MDAS13. Feelings of hopelessness MDAS14. Loss of interest MDAS 15. No pleasure MDAS 16. The future feels bleak MDAS 17. Feeling worthless MDAS 18. Poor concentration MDAS 19. Self-blame MDAS 20. Life feels meaningless MDAS 21. Feeling a failure MDAS 22. Ruminations MDAS 23. Thoughts of suicide MDAS 24. Unable to make decision Somatic domain: MDAS 25. Low energy MDAS 26. Problems with sleeping MDAS 27. Change in appetite MDAS 28. Lower sex drive MDAS 29. Feel slowed down MDAS 30. Fatigue MDAS 31. Change in weight MDAS 32. Crying MDAS 33. Agitation MDAS 34. Slowed movement MDAS 35. More pain sensitivity MDAS 36. Intestinal problems MDAS 49. Poor Memory MDAS 50. Unable to plan things MDAS 51. Feeling disorganized MDAS 52. Unable to care for myself Interpersonal domain: MDAS 37. Decrease in activities MDAS 38. Social withdrawal MDAS 39. Feeling worse than others MDAS 40. Feel a burden on others MDAS 41. Social avoidance MDAS 42. Feeling undeserving of others care MDAS 43. Hypersensitive to criticism MDAS 44. Feeling less attractive than others MDAS 45. Feel too sensitive to others MDAS 46. Feeling let down by others MDAS 47. Unable to love others MDAS 48. Aggression towards others

**Fig. 2:**
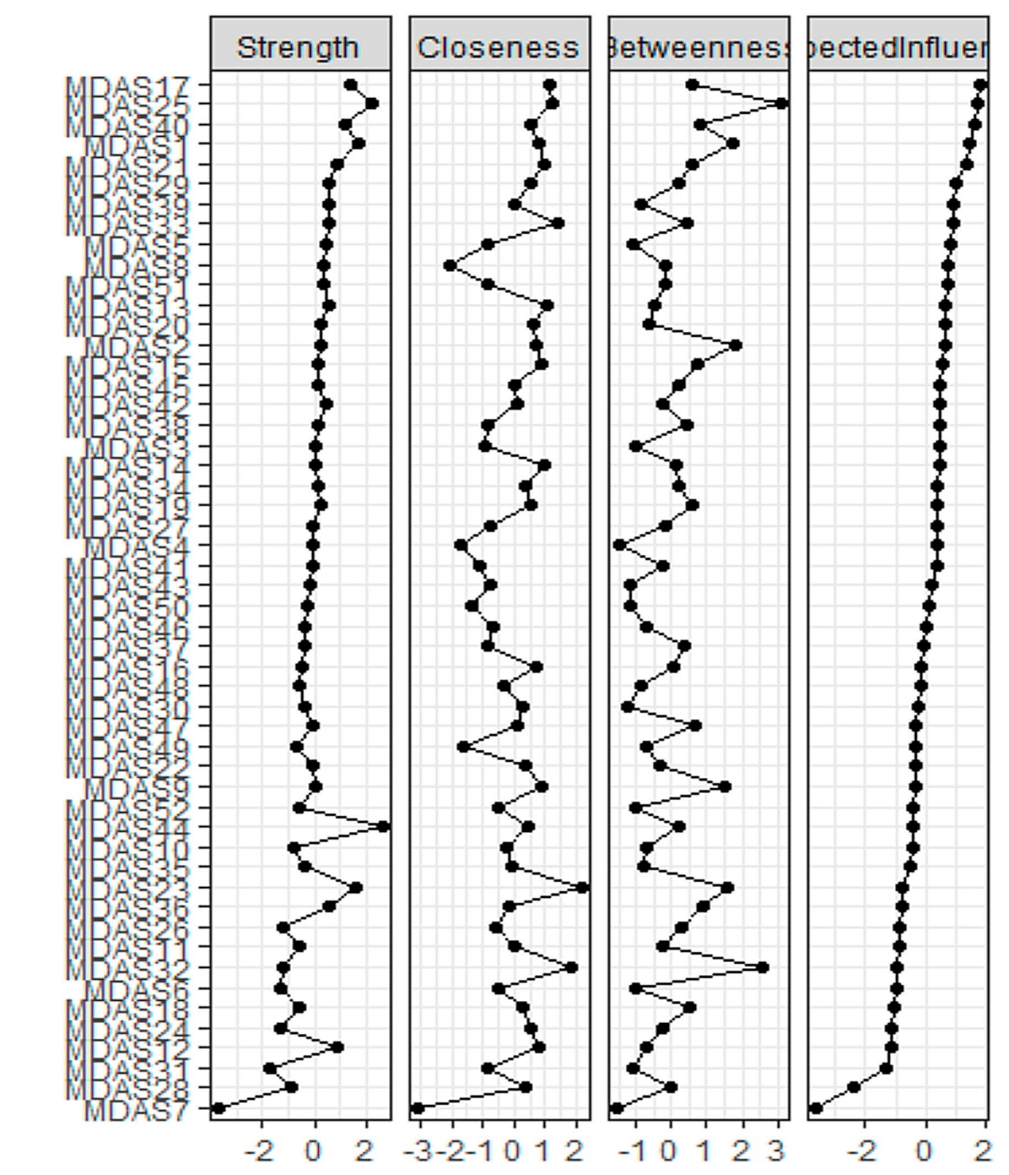
Strength Centrality for each node ordered by expected influence

The network structure for all items of MDAS is presented in Fig. 1. Figure 1 depicts the estimated EBICglasso model network of depression symptoms after multicollinearity was checked using the Goldbricker function and the highly correlated nodes were reduced. The quantitative relationships between these symptoms were evaluated with the use of a weighted adjacency matrix (Suppl. Table 2 weighted adjacency matrix). Figure 2 displays the expected influence centrality of the network’s nodes, a measure of the degree to which each node is linked to other nodes. The expected influence index demonstrated that *feeling worthless*, * low energy*, *feeling a burden on others*,* and low mood* were the central symptoms in adolescents. The supplementary figure S2 describes the distribution of the expected influence statistic for each node across the bootstrap samples (Supplementary Fig. S2).

### Network structure

The network contains 50 nodes and 683 non-zero edges. Two nodes were dropped due to multicollinearity. The network estimates demonstrated the maximum high accuracy indices for an expected influence of 0.75, i.e., after deleting up to 75% of the sample, the order of the symptoms in strength was still connected with the original one (*r* = 0.7). For detailed information about the accuracy indices of expected influence, see supplementary figure S1.

The interconnection of interpersonal symptoms of depression across Hong Kong, China, Taiwan, UK and the Netherlands.

**Fig. 3:**
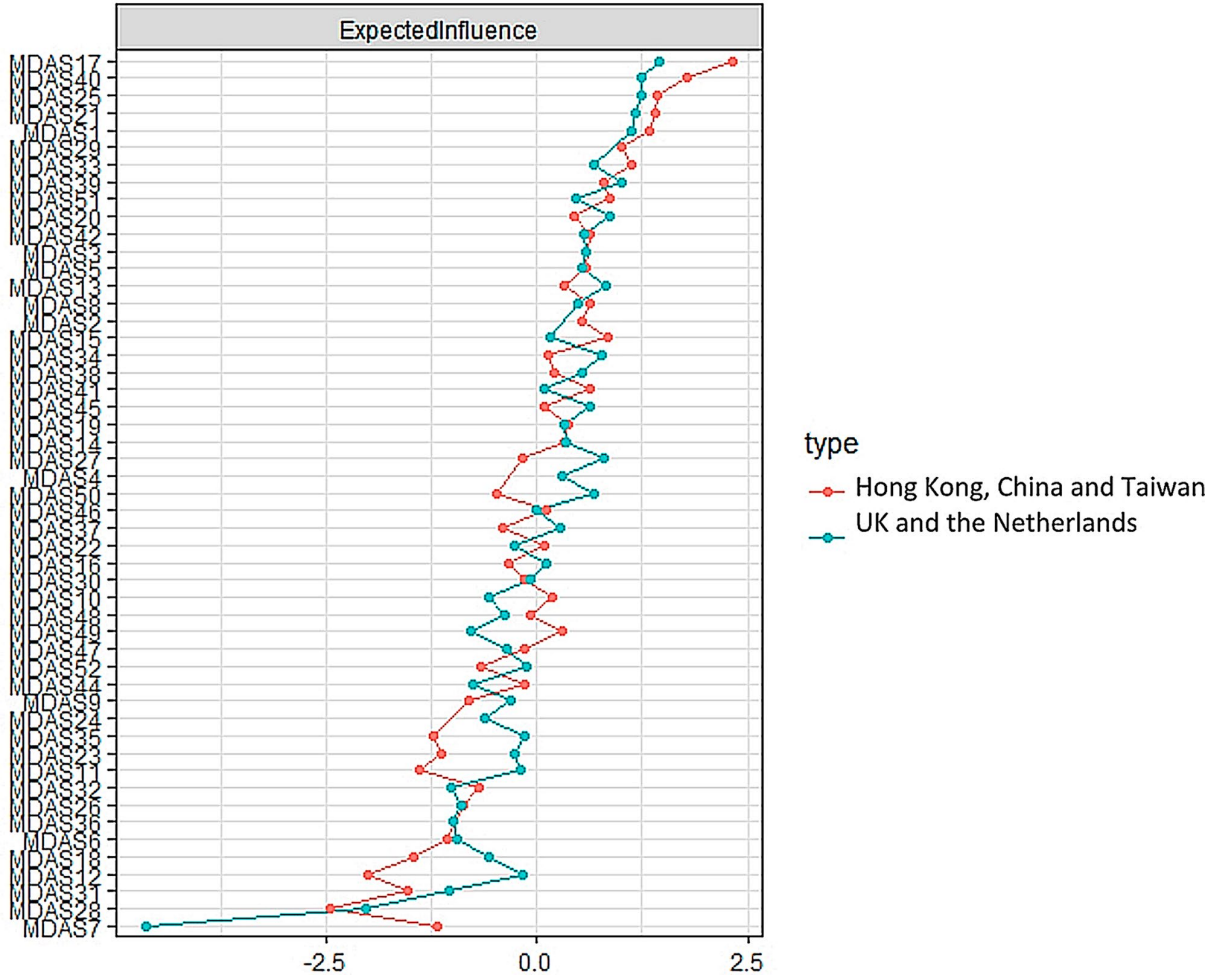
Comparison of expected influence across cultural groups

**Fig. 4:**
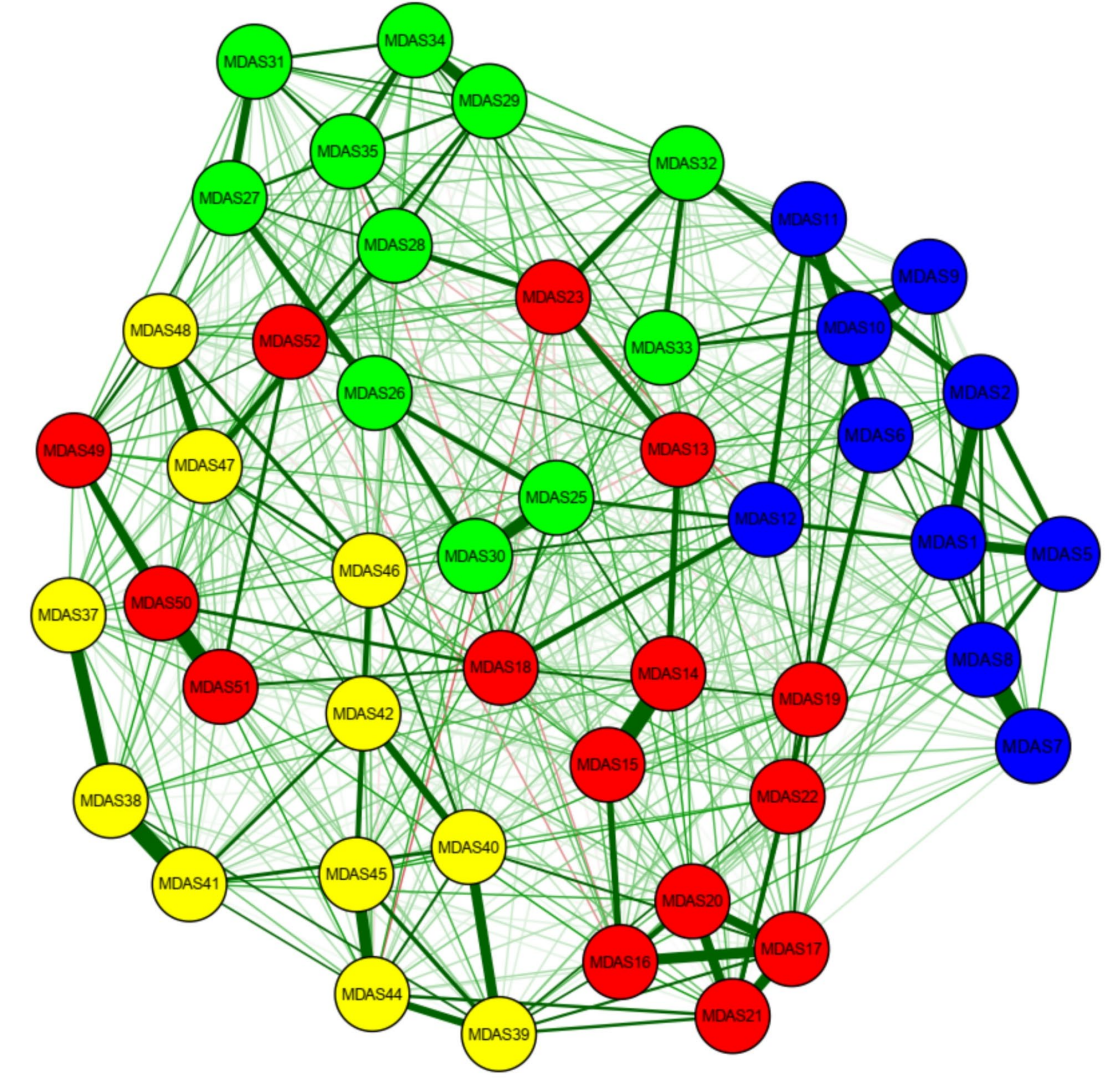
Network structure of Asian (Hong Kong, China and Taiwan) regions

Figures 3 and 4 compare network across Asian areas and European regions, showing non-significant differences in network global strength (Asian areas: 25.75 vs. European areas = 25.97; global strength difference = 0.22, *p* = 0.48), but the distribution of edge weights showed a significant difference (M = 0.17, *p* = 0.00).

**Fig. 5:**
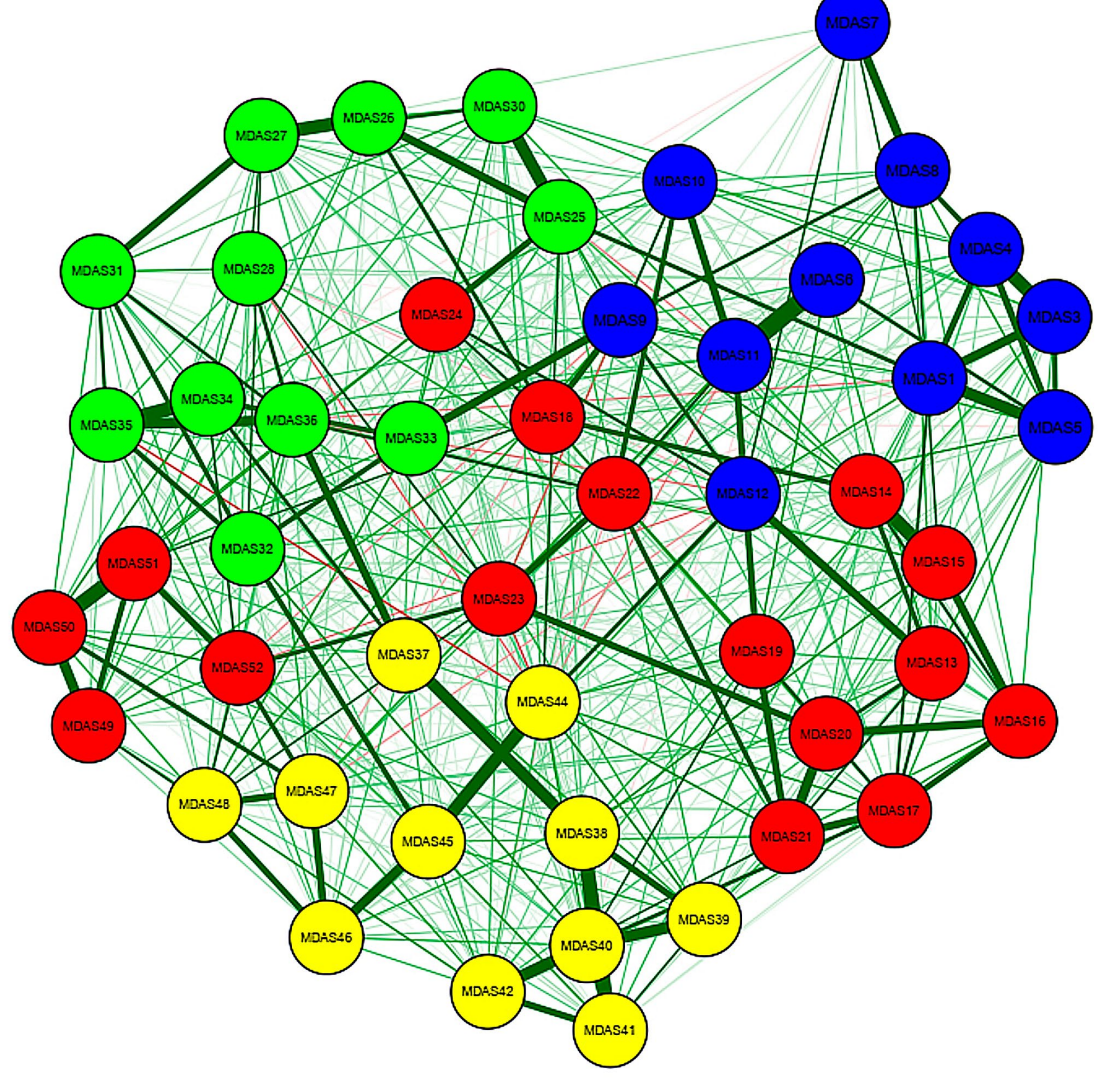
Network structure of European (UK and the Netherlands) regions

To access potential different edges between the two networks, a Bonferroni-Holm correction was used. Table 2 shows statistically significant differences (*p* < 0.001) edges between interpersonal symptoms and emotional, cognitive, and somatic symptoms across ethnic groups. For European-specific edges, Asian participants didn’t present the listed edges, while for Asian-specific edges, European participants didn’t present the listed edges. In terms of the expected influence index, a similar trend for the highest centrality node was found across ethnic groups (Fig. 5).

**Table 2:** Comparison of edge difference across Asian-region and European-region samples in terms of interpersonal symptoms

Item connection	Item description	Edge strength
**European specific edges**		
**MDAS15 and MDAS38**	No pleasure and Social withdrawal	Asian: 0 European Network: 0.07
**MDAS13 and MDAS39**	Feelings of hopelessness and Feeling worse than others	Asian: 0 European Network: 0.03
**MDAS21 and MDAS41**	Feeling a failure and Social avoidance	Asian: 0 European Network: 0.02
**MDAS2 and MDAS43**	Sadness and Hypersensitive to criticism	Asian: 0 European Network: 0.01
**MDAS32 and MDAS43**	Crying and Hypersensitive to criticism	Asian: 0 European Network: 0.02
**MDAS35 and MDAS44**	More pain sensitivity and Feeling less attractive than others	Asian: 0 European Network: -0.05
**MDAS6 and MDAS45**	Guilt and Feel too sensitive to others	Asian: 0 European Network: 0.02
**MDAS13 and MDAS45**	Feelings of hopelessness and Feel too sensitive to others	Asian: 0 European Network: 0.03
**MDAS3 and MDAS46**	Low spirits and Feeling let down by others	Asian: 0 European Network: 0.01
**Asian Specific edges**		
**MDAS6 and MDAS40**	Guilt and Feel a burden on others	Asian: 0.03 European Network: 0
**MDAS22 and MDAS40**	Ruminations and Feel a burden on others	Asian: 0.04 European Network: 0
**MDAS16 and MDAS44**	The future feels bleak and Feeling less attractive than others	Asian: 0.02 European Network: 0
**MDAS22 and MDAS46**	Ruminations and Feeling let down by others	Asian: 0.05 European Network: 0
**MDAS1 and MDAS47**	Low mood and Unable to love others	Asian: 0.004 European Network: 0
**MDAS3 and MDAS47**	Low spirits and Unable to love others	Asian: 0.02 European Network: 0
**MDAS5 and MDAS47**	Sad mood and Unable to love others	Asian: 0.003 European Network: 0
**MDAS34 and MDAS48**	Slowed movement and Aggression towards others	Asian: 0.06 European Network: 0

## Discussion

The current study is the first to incorporate interpersonal depression symptoms into a mixture of symptom patterns in adolescents using the network approach in a large heterogeneous sample. Their manifestation of depression was compared in symptom level across Hong Kong, China, and Taiwan, UK and the Netherlands. The current study bridges the knowledge gap of previous network studies using PHQ-9 [30], which overlooked the interpersonal symptoms of depression. It also adds to the discussion of the heterogeneity of MDD criteria [58] towards a more personalized diagnosis and treatment [59] using the more predictable centrality index of expected influence. The network model identified *feeling worthless*,* low energy*,* feeling a burden on others*,* and low mood* as central traits. The results are consistent with previous literature on interpersonal sensitivity in adolescents. For instance, it has been recorded that interpersonal interactions affect adolescents’ sense of self-esteem due to their growing capacity to build meaningful psychological relationships with others and the opportunity to learn about themselves and their own functioning in particular circumstances [60, 61]. Simultaneously, research has revealed that relationships may be a prevalent cause of stress throughout adolescence [62]. Adolescents are particularly sensitive to their social environments [63]. The node *Feeling like a burden* may result from perceived or real changes in their relationships with family and friends, often because they are unable to contribute to or participate in social situations as they believe they should. Despite the abundant evidence of interpersonal stressors in adolescents [64], the current study identified the important role of interpersonal symptoms in depression in adolescents and the predictive role for future treatments. In their network study, Mullarkey et al. [32] identified *self-hatred*,* loneliness*,* sadness*,* and pessimism* as central features of depression. In comparison, *Low mood* in the present study corresponds with *sadness*. Both *self-hatred* in their study *and feeling worthless* in the present study primarily encompass the individual’s self-image and perspective. They might have a substantial negative impact on interpersonal relationships by influencing individuals’ self-perception in social situations. Experiencing a sense of self-hatred and worthlessness might cause individuals to isolate themselves from social connections believing that they do not contribute any value to their relationships [65]. On the other hand, *feeling like a burden* from the present study entails one’s self-perception in relation to others. It can induce individuals to distance themselves from intimate connections to avoid burdening others with their presence or needs, resulting in isolation and a deterioration of social support networks.

The present study is also the first to investigate the differences in symptom connection between Hong Kong, China, and Taiwan, UK and the Netherlands in depressive symptoms using a comprehensive MDAS symptom profile. The study investigated the role of interpersonal symptoms in relation to other depressive symptoms, their potential role in depression, and the unique connections within each ethnic group. The result supported the differences in network structure across Asian regions (Hong Kong, China and Taiwan) and European (UK and the Netherlands) regions. In terms of how interpersonal symptoms are related to the emotional, cognitive, and somatic domains, Hong Kong, China and Taiwan and UK and Dutch participants demonstrated variations in the linkage. An Asian-specific connection, for example, is *feeling less attractive than others* and its relation to the *future feels bleak*. This is in line with previous studies showing that a thinner physique is more preferable in Chinese culture and that Chinese women perceive their body image more negatively [66]. In terms of the connection between being *unable to love others* and emotional depressive symptoms in Hong Kong, China and Taiwan rather than UK and Dutch participants, it might reflect a more collectivistic attitude towards contributing to society in Asian populations. The linkage aligned with a previous study of the correlation between adherence to Asian cultural values and the sense of group identity and self-critical perfectionism in Asian American and Asian foreign college students [67]. Moreover, the Asian-specific linkage of *feeling a burden on others* with *guilt and rumination* collides with the Asian value of being useful to family and society, taking into account the interdependence of self-identity in family and interpersonal relationships. It is also consistent with self-exertion that holds moral value in Eastern cultures [68] and more extensive interpersonal networks in their work, family, and school lives compared to their counterparts in the US [69]. In particular, when individuals identified specifically with Asian ethnicity, their normative expectations of collectivism would predict the Asian pattern of depressive symptoms. Finally, *slow movement* and *aggression towards others* were identified as an Asian-specific linkage. Psychomotor retardation in adolescents has been well-documented in current literature [70]. However, Asian adolescents may feel misunderstood by their community or family members who might perceive their lack of activity as intentional or avoidant. This misunderstanding might lead to frustration and aggressive reactions to express distress or defend oneself against perceived criticism or pressure. In many Asian cultures, there is substantial pressure to perform and meet high societal and familial expectations [71, 72]. Those experiencing slowed movement due to depression may find themselves increasingly unable to meet these expectations, leading to internal stress and eventual aggression as a response to overwhelming pressure and a lack of other coping mechanisms.

However, in European-specific linkages, a different pattern of culturally specific connections was reported. In Western societies, the relationship between interpersonal symptoms and other characteristics of depression is primarily rooted in heightened sensitivity within social connections. For example, European-specific linkages included hypersensitivity to criticism, sadness, and crying, as well as guilt and hopeless feelings well established in the interpersonal model of depression.

The findings from the current study have several important implications for clinical practice. Practitioners could pay attention to the identified central traits and consider them potential focal points in therapeutic interventions. Tailoring treatments to target these core symptoms could improve outcomes by addressing the most influential aspects of the patient’s depression. The differences in network structures between Hong Kong, China, and Taiwan, UK and the Netherlands adolescents suggest that cultural background significantly influences how depression manifests. Clinicians should adopt a culturally sensitive approach, recognizing that interpersonal relationships and cultural values play a crucial role in the experience of depression. For example, in Asian adolescents, feelings of being a burden may be more prominent due to cultural values of interdependence and collectivism. Understanding these cultural nuances can guide more effective, culturally congruent therapeutic approaches. Programs such as interpersonal psychotherapy (IPT) or family therapy might be particularly beneficial for Asian adolescents, addressing key issues such as communication skills, conflict resolution, and emotional expression within relationships. Finally, interpersonal symptoms’ central role in the depression network could facilitate earlier screening and intervention in clinical settings for a better outcome.

### Limitations

The study encompasses a few limitations. First, the cross-sectional design impedes the study of the causality of variables and would affect the implications of addressing high-centrality nodes as potential clinical targets. Future longitudinal studies should be implemented for better clinical use. Second, previous research has found that age, education level, and marital status impact the epidemiology and clinical aspects of depression [73, 74]. However, due to the homogeneity of the present population in terms of age and education level, the current sample did not account for these variables. Furthermore, the interpretation focused on strength centrality instead of betweenness and closeness centrality. While the centrality stability for betweenness and closeness centrality did not meet the threshold requirement, the interpretation aligned with the theories of LF Bringmann, T Elmer, S Epskamp, RW Krause, D Schoch, M Wichers, JTW Wigman and E Snippe [75]. These theories suggest that betweenness and closeness centrality are particularly unsuitable for most psychological networks, as they rely on strong assumptions such as the shortest route, which may or may not apply, not only in psychological networks but also in social and brain networks. Future studies yielding better centrality stability for all centrality indices could compare the results from all indices. Finally, these regions in the study where the sample was recruited from do not represent the entirety of their respective geographic areas. Hence future studies that include more areas could provide a more detailed picture of depressive symptoms across Eastern and Western cultures.

## Conclusions

This research is the first to use the network technique to include interpersonal symptoms of depression in a diverse sample of adolescents. Interpersonal symptoms of *feeling worthless*,* low energy*,* feeling a burden on others*,* and low mood* are central features in addition to low energy and low mood in adolescents. This research indicated that, compared to their UK and Dutch counterparts, interpersonal symptoms link to emotional, cognitive, and somatic symptoms across ethnic groups in a unique way, corresponding to cultural characteristics. This suggests significant clinical and research implications, necessitating a review of future intervention directions to align with the findings.

## Electronic supplementary material

Below is the link to the electronic supplementary material.

Supplementary Material 1

Supplementary Material 2

Supplementary Material 3

Supplementary Material 4

## Data Availability

The datasets analyzed during the current study are not publicly available due to the requirement by ethnics committee but are available from the primary corresponding author Dr H. N Cheung on request.

## Abbreviations

MDD: Major Depressive Disorder
MDAS: Multidimensional Depression Assessment Scale
GGM: Gaussian Graphical Model
PHQ-9: Patient Health Questionnaire-9
DSM: Diagnostic and Statistical Manual of Mental Disorders
EBICglasso: Extended Bayesian Information Criterion graphical least absolute shrinkage and selection operator
LASSO: Least Absolute Shrinkage and Selection Operator
NCT: Network Comparison Test
SD: Standard Deviation
CS: Correlation Stability
IPT: Interpersonal psychotherapy

## References

[1] Depression. fact sheet [http://who.int/mediacentre/factsheets/fs369/en/

[2] Costello EJ, Egger H, Angold A. 10-year research update review: the epidemiology of child and adolescent psychiatric disorders: I. methods and public health burden. J Am Acad Child Adolesc Psychiatry. 2005;44(10):972–86. 16175102 10.1097/01.chi.0000172552.41596.6f

[3] Young CB, Fang DZ, Zisook S. Depression in Asian–American and Europeanundergraduate students. J Affect Disord. 2010;125(1):379–82. 20303181 10.1016/j.jad.2010.02.124

[4] Ahn H, Weaver M, Lyon D, Choi E, Fillingim RB. Depression and Pain in Asian and White americans with knee osteoarthritis. J Pain. 2017;18(10):1229–36. 28619697 10.1016/j.jpain.2017.05.007 PMC5661986

[5] Flores MW, Sharp A, Carson NJ, Cook BL. Estimates of major depressive disorder and treatment among adolescents by race and ethnicity. JAMA Pediatr. 2023;177(11):1215–23. 37812424 10.1001/jamapediatrics.2023.3996 PMC10562990

[6] Monroe SM, Anderson S. Depression: the shroud of heterogeneity. Curr Dir Psychol Sci. 2015;24(3):227–31.

[7] Sobański JA, Klasa K, Dembińska E, Mielimąka M, Citkowska-Kisielewska A, Jęda P, Rutkowski K. Central psychological symptoms from a network analysis of patients with anxiety, somatoform or personality disorders before psychotherapy. J Affect Disord. 2023;339:1–21. 37399849 10.1016/j.jad.2023.06.040

[8] Klerman GL, Weissman MM. Interpersonal psychotherapy of depression: a brief, focused, specific strategy. Jason Aronson, Incorporated; 1994.

[9] Joiner TE Jr. A test of interpersonal theory of depression in youth psychiatric inpatients. J Abnorm Child Psychol. 1999;27(1):77–85. 10197408 10.1023/a:1022666424731

[10] Rose-Clarke K, Hassan E, Bk P, Magar J, Devakumar D, Luitel NP, Verdeli H, Kohrt BA. A cross-cultural interpersonal model of adolescent depression: a qualitative study in rural Nepal. Soc Sci Med. 2021;270:113623. 33461033 10.1016/j.socscimed.2020.113623 PMC7895817

[11] Coyne JC. Toward an interactional description of depression. In., vol. 39. US: Guilford Publications; 1976: 28–40. 10.1080/00332747.1976.11023874 1257353

[12] Coyne JC. Depression and the response of others. J Abnorm Psychol. 1976;85:186–93. 1254779 10.1037//0021-843x.85.2.186

[13] Lipsitz JD, Markowitz JC. Mechanisms of change in interpersonal therapy (IPT). Clin Psychol Rev. 2013;33(8):1134–47. 24100081 10.1016/j.cpr.2013.09.002 PMC4109031

[14] Cheung HN, Power MJ. The development of a New Multidimensional Depression Assessment Scale: preliminary results. Clin Psychol Psychother. 2012;19(2):170–8. 22336997 10.1002/cpp.1782

[15] Cheung HN, Williams JM, Chan YS, Chan SWY. Measurement invariance of the Multidimensional Depression Assessment Scale (MDAS) across gender and ethnic groups of Asian, caucasian, black, and hispanic. J Affect Disord. 2022;308:221–8. 35429539 10.1016/j.jad.2022.04.035

[16] Umaña-Taylor AJ, Updegraff KA. Latino adolescents’ Mental Health: exploring the interrelations among discrimination, ethnic identity, Cultural Orientation, Self‐esteem, and depressive symptoms. J Adolesc 2006. 10.1016/j.adolescence.2006.08.002 17056105

[17] Cohen JR, Spiro CN, Young JF, Gibb BE, Hankin BL, Abela JRZ. Interpersonal risk profiles for Youth Depression: a Person-Centered, Multi-wave, Longitudinal Study. J Abnorm Child Psychol 2015. 10.1007/s10802-015-0023-x PMC4609227 25907029

[18] Kleinman A. Neurasthenia and Depression: a study of somatization and culture in China. Cult Med Psychiatry 1982. 10.1007/BF00051427 7116909

[19] Fukita S, Kawasaki H, Yamasaki S. Comprehensive Analysis of Depression-related factors among Middle-aged residents in Japan, an Eastern Culture. Medicine; 2021. 10.1097/MD.0000000000025735 PMC8133093 34106600

[20] Haroz EE, Ritchey M, Bass JK, Kohrt BA, Augustinavicius J, Michalopoulos L, Burkey MD, Bolton P. How is depression experienced around the world? A systematic review of qualitative literature. Soc Sci Med. 2017;183:151–62. 28069271 10.1016/j.socscimed.2016.12.030 PMC5488686

[21] Chentsova-Dutton YE, Ryder AG, Tsai J. Understanding depression across cultural contexts. Handbook of depression. 3rd ed. New York, NY, US: Guilford Press; 2014. pp. 337–54.

[22] Stange JP, Hamilton JL, Abramson LY, Alloy LB. A vulnerability-stress examination of response styles theory in adolescence: stressors, sex differences, and Symptom specificity. J Clin Child Adolesc Psychol. 2014;43(5):813–27. 23829270 10.1080/15374416.2013.812037 PMC3825810

[23] Fried EI, Nesse RM. Depression sum-scores don’t add up: why analyzing specific depression symptoms is essential. BMC Med. 2015;13(1):72. 25879936 10.1186/s12916-015-0325-4 PMC4386095

[24] Fried EI, Nesse RM. Depression is not a consistent syndrome: an investigation of unique symptom patterns in the STAR*D study. J Affect Disord. 2015;172:96–102. 25451401 10.1016/j.jad.2014.10.010 PMC4397113

[25] Insel TR. The NIMH Research Domain Criteria (RDoC) Project: Precision Medicine for Psychiatry. Am J Psychiatry. 2014;171(4):395–7. 24687194 10.1176/appi.ajp.2014.14020138

[26] Borsboom D. A network theory of mental disorders. World Psychiatry. 2017;16(1):5–13. 28127906 10.1002/wps.20375 PMC5269502

[27] Epskamp S, Kruis J, Marsman M. Estimating psychopathological networks: be careful what you wish for. PLoS ONE. 2017;12(6):e0179891. 28644856 10.1371/journal.pone.0179891 PMC5482475

[28] Robinaugh DJ, Millner AJ, McNally RJ. Identifying highly influential nodes in the complicated grief network. J Abnorm Psychol. 2016;125(6):747–57. 27505622 10.1037/abn0000181 PMC5060093

[29] Borsboom D, Cramer AO. Network analysis: an integrative approach to the structure of psychopathology. Annu Rev Clin Psychol. 2013;9:91–121. 23537483 10.1146/annurev-clinpsy-050212-185608

[30] Cheung T, Jin Y, Lam S, Su Z, Hall BJ, Xiang Y-T, Suen LKP, Chan S, Ho HSW, Lam KBH, et al. Network analysis of depressive symptoms in Hong Kong residents during the COVID-19 pandemic. Translational Psychiatry. 2021;11(1):460. 34489416 10.1038/s41398-021-01543-z PMC8419676

[31] Crosnoe R, Johnson MK. Research on adolescence in the twenty-first century. Ann Rev Sociol. 2011;37:439–60. 29167597 10.1146/annurev-soc-081309-150008 PMC5695926

[32] Mullarkey MC, Marchetti I, Beevers CG. Using Network Analysis to identify central symptoms of adolescent depression. J Clin Child Adolesc Psychol. 2019;48(4):656–68. 29533089 10.1080/15374416.2018.1437735 PMC6535368

[33] Moradi S, Falsafinejad MR, Delavar A, Rezaeitabar V, Borj’ali A, Aggen SH, Kendler KS. Network modeling of major depressive disorder symptoms in adult women. Psychol Med. 2023;53(12):5449–58. 36004799 10.1017/S0033291722002604

[34] St Quinton T, Stain HJ. A Network Approach to Depressive disorders. J Rational-Emot Cognitive-Behav Ther. 2020;38(1):1–13.

[35] Tang H, Quertermous T, Rodriguez B, Kardia SL, Zhu X, Brown A, Pankow JS, Province MA, Hunt SC, Boerwinkle E, et al. Genetic structure, self-identified race/ethnicity, and confounding in case-control association studies. Am J Hum Genet. 2005;76(2):268–75. 15625622 10.1086/427888 PMC1196372

[36] Mersha TB, Abebe T. Self-reported race/ethnicity in the age of genomic research: its potential impact on understanding health disparities. Hum Genomics. 2015;9(1):1. 25563503 10.1186/s40246-014-0023-x PMC4307746

[37] Bowins B. Depression: discrete or continuous? Psychopathology. 2015;48(2):69–78. 25531962 10.1159/000366504

[38] Pauszek JR, Sztybel P, Gibson BS. Evaluating Amazon’s mechanical Turk for psychological research on the symbolic control of attention. Behav Res Methods. 2017;49(6):1969–83. 28127682 10.3758/s13428-016-0847-5

[39] Cheung HN, Williams JM, Chan SWY. A cultural validation of the Chinese version of multidimensional depression assessment scale (MDAS) in clinically depressed patients in Inner Mongolia. Curr Psychol. 2022;41(9):5948–58.

[40] Darharaj M, Habibi M, Power MJ, Farzadian F, Rahimi M, Kholghi H, Kazemitabar M. Inpatients with major depressive disorder: psychometric properties of the new Multidimensional Depression Scale. Asian J Psychiatr. 2016;24:103–9. 27931890 10.1016/j.ajp.2016.08.018

[41] Darharaj M, Habibi M, Power MJ, Pirirani S, Tehrani F. Factor structure and psychometric properties of the new multidimensional depression scale in a non-clinical sample. Clin Psychol. 2018;22(1):63–71.

[42] Habibi Asgarabad M, Yegaei P, Ho W, Cheung H. The gender invariance of Multidimensional Depression Assessment Scale in adolescents. J Psychopathol Behav Assess. 2023;45:1–10.37691858

[43] Tyupa S. A theoretical framework for back-translation as a quality assessment tool. 2011, 7:35–46.

[44] Fan J, Liao Y, Liu H. An overview of the estimation of large covariance and precision matrices. Econometrics J. 2016;19(1):C1–32.

[45] Epskamp S, Borsboom D, Fried EI. Estimating psychological networks and their accuracy: a tutorial paper. Behav Res Methods. 2018;50(1):195–212. 28342071 10.3758/s13428-017-0862-1 PMC5809547

[46] Friedman J, Hastie T, Tibshirani R. Sparse inverse covariance estimation with the graphical lasso. Biostatistics. 2007;9(3):432–41. 18079126 10.1093/biostatistics/kxm045 PMC3019769

[47] Chen J, Chen Z. Extended bayesian information criteria for model selection with large model spaces. Biometrika. 2008;95(3):759–71.

[48] Jones P. Networktools: Tools for identifying important nodes in networks. [Computer software]. In. https://CRAN.R-project.org/package=networktools; 2018.

[49] Xu Y, Yang X. A Novel Chaotic Neural Network With Anti-Trigonometric Function Self-Feedback. 2010.

[50] Opsahl T, Agneessens F, Skvoretz J. Node centrality in weighted networks: generalizing degree and shortest paths. Social Networks. 2010;32(3):245–51.

[51] Spiller TR, Levi O, Neria Y, Suarez-Jimenez B, Bar-Haim Y, Lazarov A. On the validity of the centrality hypothesis in cross-sectional between-subject networks of psychopathology. BMC Med. 2020;18(1):297. 33040734 10.1186/s12916-020-01740-5 PMC7549218

[52] Fruchterman TMJ, Reingold EM. Graph drawing by force-directed placement. Software: Pract Experience. 1991;21(11):1129–64.

[53] Epskamp S, Cramer AOJ, Waldorp LJ, Schmittmann VD, Borsboom D. Qgraph: network visualizations of relationships in Psychometric Data. J Stat Softw. 2012;48(4):1–18.

[54] Epskamp S, Fried EI. bootnet: Bootstrap methods for various network estimation routines. In.: CRAN.r-project.org.; 2015.

[55] van Borkulo CD, van Bork R, Boschloo L, Kossakowski JJ, Tio P, Schoevers RA, Borsboom D, Waldorp LJ. Comparing network structures on three aspects: A permutation test. *Psychological Methods* 2022:No Pagination Specified-No Pagination Specified. 10.1037/met0000476 35404628

[56] Wang Y, Hu Z, Feng Y, Wilson A, Chen R. Changes in network centrality of psychopathology symptoms between the COVID-19 outbreak and after peak. Mol Psychiatry. 2020;25(12):3140–9. 32929212 10.1038/s41380-020-00881-6 PMC7488637

[57] van Borkulo CD, Epskamp S. IsingFit: fitting ising models using the ELasso Method; Version 0.3.1. With contributions from Alexander Robitzsch. In.: [IsingSampler]. CRAN.r-project.org.; 2016.

[58] Kim YK, Park SC. An alternative approach to future diagnostic standards for major depressive disorder. Prog Neuropsychopharmacol Biol Psychiatry. 2021;105:110133. 33049324 10.1016/j.pnpbp.2020.110133

[59] Insel TR. Next-generation treatments for mental disorders. Sci Transl Med. 2012;4(155):ps155119–155119. 10.1126/scitranslmed.3004873 23052292

[60] Compare A, Zarbo C, Manzoni GM, Castelnuovo G, Baldassari E, Bonardi A, Callus E, Romagnoni C. Social support, depression, and heart disease: a ten year literature review. Front Psychol. 2013;4:384. 23847561 10.3389/fpsyg.2013.00384 PMC3696881

[61] Buonomo I, Fiorilli C, Geraci MA, Pepe A. Temperament and social-emotional difficulties: the Dark side of learning disabilities. J Genet Psychol. 2017;178(3):193–206. 28402224 10.1080/00221325.2017.1304890

[62] Compas BE, Jaser SS, Bettis AH, Watson KH, Gruhn MA, Dunbar JP, Williams E, Thigpen JC. Coping, emotion regulation, and psychopathology in childhood and adolescence: a meta-analysis and narrative review. Psychol Bull. 2017;143(9):939–91. 28616996 10.1037/bul0000110 PMC7310319

[63] Blakemore S-J, Mills KL. Is adolescence a sensitive period for Sociocultural Processing? Ann Rev Psychol. 2014;65(1):187–207. 24016274 10.1146/annurev-psych-010213-115202

[64] Chen R, Peng K, Liu J, Wilson A, Wang Y, Wilkinon MR, Wen S, Cao X, Lu J. Interpersonal trauma and risk of Depression among adolescents: the Mediating and moderating Effect of Interpersonal Relationship and Physical Exercise. Front Psychiatry. 2020;11:194. 32351408 10.3389/fpsyt.2020.00194 PMC7174748

[65] Ypsilanti A. Lonely but avoidant—the unfortunate juxtaposition of loneliness and self-disgust. Palgrave Commun. 2018;4(1):144.

[66] Stojcic I, Dong X, Ren X. Body image and sociocultural predictors of body image dissatisfaction in Croatian and Chinese women. Front Psychol 2020, 11. 10.3389/fpsyg.2020.00731 PMC7218091 32435214

[67] Suh HN, Pigott TD, Rice KG, Davis DE, Andrade AC. Meta-analysis of the relationship between self-critical perfectionism and depressive symptoms: comparison between Asian American and Asian International College Students. J Couns Psychol 2023. 10.1037/cou0000653 36521120

[68] Li J. Cultural foundations of Learning: East and West. Cambridge: Cambridge University Press; 2012.

[69] Hu P, Tan Y. The comparison of Chinese and American Interpersonal relationships. In: 2013/10 2013. Atlantis; 2013. pp. 1118–20.

[70] Korczak DJ, Westwell-Roper C, Sassi R. Diagnosis and management of depression in adolescents. CMAJ. 2023;195(21):E739–46. 37247881 10.1503/cmaj.220966 PMC10228578

[71] Wei M, Liu S, Ko SY, Wang C, Du Y. Impostor feelings and psychological distress among Asian americans: interpersonal shame and Self-Compassion. Couns Psychol. 2020;48(3):432–58.

[72] Lui PP. Intergenerational Cultural Conflict, Mental Health, and Educational outcomes among Asian and Latino/a americans: qualitative and Meta-Analytic Review. Psychol Bull. 2015;141(2):404–46. 25528344 10.1037/a0038449

[73] Gan Z, Li Y, Xie D, Shao C, Yang F, Shen Y, Zhang N, Zhang G, Tian T, Yin A, et al. The impact of educational status on the clinical features of major depressive disorder among Chinese women. J Affect Disord. 2012;136(3):988–92. 21824664 10.1016/j.jad.2011.06.046 PMC3314924

[74] Buckman JEJ, Saunders R, Stott J, Arundell LL, O’Driscoll C, Davies MR, Eley TC, Hollon SD, Kendrick T, Ambler G, et al. Role of age, gender and marital status in prognosis for adults with depression: an individual patient data meta-analysis. Epidemiol Psychiatr Sci. 2021;30:e42. 34085616 10.1017/S2045796021000342 PMC7610920

[75] Bringmann LF, Elmer T, Epskamp S, Krause RW, Schoch D, Wichers M, Wigman JTW, Snippe E. What do centrality measures measure in psychological networks? J Abnorm Psychol. 2019;128(8):892–903. 31318245 10.1037/abn0000446

