# Supplementary material for: Interpersonal symptoms in adolescence depression across Asian and European regions: a network approach
Source: BMC Psychiatry. 2024 Oct 22;24:713. doi: 10.1186/s12888-024-06161-9 (PMC11515707; doi:10.1186/s12888-024-06161-9)
Supplement: Supplementary file 1 — Supplementary Material 1 [file 12888_2024_6161_MOESM1_ESM.docx]

Supplementary Table 1. The mean, standard deviation, skewness, and kurtosis of 52 MDAS items.

|  | Mean | Std. Deviation | Skewness | | Kurtosis | |
| --- | --- | --- | --- | --- | --- | --- |
|  | Statistic | Statistic | Statistic | Std. Error | Statistic | Std. Error |
| MDAS1 | 2.43 | 1.119 | .450 | .030 | -.575 | .059 |
| MDAS2 | 2.39 | 1.098 | .554 | .030 | -.424 | .059 |
| MDAS3 | 2.47 | 1.142 | .458 | .030 | -.642 | .059 |
| MDAS4 | 2.36 | 1.138 | .572 | .030 | -.479 | .059 |
| MDAS5 | 2.41 | 1.132 | .520 | .030 | -.535 | .059 |
| MDAS6 | 2.26 | 1.152 | .685 | .030 | -.392 | .059 |
| MDAS7 | 2.50 | 1.115 | .425 | .030 | -.592 | .059 |
| MDAS8 | 2.42 | 1.089 | .515 | .030 | -.413 | .059 |
| MDAS9 | 2.48 | 1.194 | .423 | .030 | -.769 | .059 |
| MDAS10 | 2.28 | 1.136 | .640 | .030 | -.410 | .059 |
| MDAS11 | 2.16 | 1.112 | .794 | .030 | -.138 | .059 |
| MDAS12 | 2.63 | 1.255 | .358 | .030 | -.885 | .059 |
| MDAS13 | 2.27 | 1.222 | .694 | .030 | -.516 | .059 |
| MDAS14 | 2.44 | 1.211 | .522 | .030 | -.673 | .059 |
| MDAS15 | 2.37 | 1.181 | .562 | .030 | -.592 | .059 |
| MDAS16 | 2.49 | 1.285 | .483 | .030 | -.855 | .059 |
| MDAS17 | 2.39 | 1.256 | .583 | .030 | -.709 | .059 |
| MDAS18 | 2.58 | 1.244 | .392 | .030 | -.840 | .059 |
| MDAS19 | 2.43 | 1.214 | .536 | .030 | -.665 | .059 |
| MDAS20 | 2.34 | 1.270 | .652 | .030 | -.663 | .059 |
| MDAS21 | 2.38 | 1.252 | .598 | .030 | -.677 | .059 |
| MDAS22 | 2.27 | 1.191 | .690 | .030 | -.452 | .059 |
| MDAS23 | 1.92 | 1.147 | 1.106 | .030 | .236 | .059 |
| MDAS24 | 2.53 | 1.230 | .420 | .030 | -.798 | .059 |
| MDAS25 | 2.51 | 1.227 | .464 | .030 | -.748 | .059 |
| MDAS26 | 2.45 | 1.263 | .556 | .030 | -.721 | .059 |
| MDAS27 | 2.31 | 1.208 | .664 | .030 | -.519 | .059 |
| MDAS28 | 2.02 | 1.161 | .999 | .030 | .083 | .059 |
| MDAS29 | 2.21 | 1.156 | .732 | .030 | -.339 | .059 |
| MDAS30 | 2.52 | 1.222 | .420 | .030 | -.778 | .059 |
| MDAS31 | 2.16 | 1.154 | .786 | .030 | -.258 | .059 |
| MDAS32 | 2.24 | 1.173 | .730 | .030 | -.372 | .059 |
| MDAS33 | 2.35 | 1.186 | .572 | .030 | -.594 | .059 |
| MDAS34 | 2.13 | 1.140 | .827 | .030 | -.167 | .059 |
| MDAS35 | 2.10 | 1.139 | .843 | .030 | -.175 | .059 |
| MDAS36 | 2.07 | 1.149 | .915 | .030 | -.048 | .059 |
| MDAS37 | 2.32 | 1.198 | .608 | .030 | -.587 | .059 |
| MDAS38 | 2.33 | 1.240 | .616 | .030 | -.661 | .059 |
| MDAS39 | 2.38 | 1.248 | .572 | .030 | -.721 | .059 |
| MDAS40 | 2.36 | 1.269 | .609 | .030 | -.701 | .059 |
| MDAS41 | 2.37 | 1.272 | .595 | .030 | -.729 | .059 |
| MDAS42 | 2.28 | 1.244 | .705 | .030 | -.538 | .059 |
| MDAS43 | 2.42 | 1.258 | .543 | .030 | -.747 | .059 |
| MDAS44 | 2.56 | 1.336 | .448 | .030 | -.964 | .059 |
| MDAS45 | 2.42 | 1.262 | .547 | .030 | -.744 | .059 |
| MDAS46 | 2.33 | 1.224 | .649 | .030 | -.549 | .059 |
| MDAS47 | 2.20 | 1.199 | .769 | .030 | -.382 | .059 |
| MDAS48 | 2.20 | 1.190 | .790 | .030 | -.305 | .059 |
| MDAS49 | 2.28 | 1.217 | .664 | .030 | -.550 | .059 |
| MDAS50 | 2.26 | 1.187 | .682 | .030 | -.462 | .059 |
| MDAS51 | 2.36 | 1.208 | .572 | .030 | -.635 | .059 |
| MDAS52 | 2.11 | 1.176 | .868 | .030 | -.192 | .059 |
