# Supplementary figures and images for: Interpersonal symptoms in adolescence depression across Asian and European regions: a network approach
Source: BMC Psychiatry. 2024 Oct 22;24:713. doi: 10.1186/s12888-024-06161-9 (PMC11515707; doi:10.1186/s12888-024-06161-9)

Supplementary Figure S1 accuracy index of expected influence


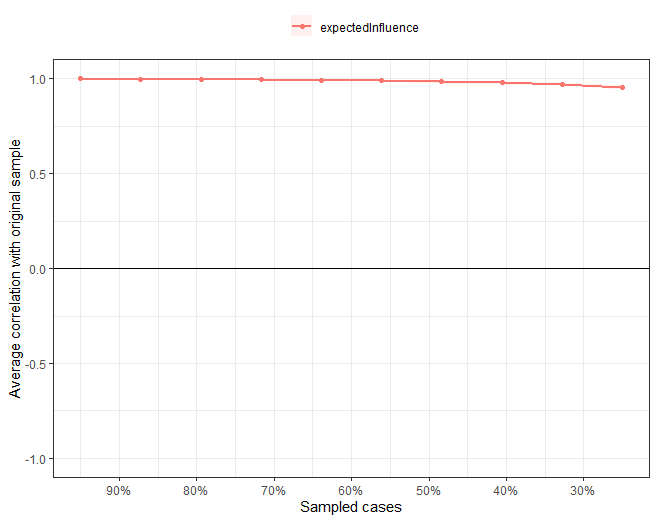

Supplement: Supplementary file 3 — Supplementary Material 3 [file 12888_2024_6161_MOESM3_ESM.docx]
